# PyKleeBarcode: Enabling representation of the whole animal kingdom in information space

**DOI:** 10.1371/journal.pone.0286314

**Published:** 2023-06-02

**Authors:** Wandrille Duchemin, David S. Thaler

**Affiliations:** 1 sciCORE Center for Scientific Computing, University of Basel, Basel, Switzerland; 2 Biozentrum, University of Basel, Basel, Switzerland; 3 Program for the Human Environment, Rockefeller University, New York, NY, United States of America; AbbVie Inc, UNITED STATES

## Abstract

As biological sequence databases continue growing, so do the insight that they promise to shed on the shape of the genetic diversity of life. However, to fulfil this promise the software must remain usable, be able to accommodate a large amount of data and allow use of modern high performance computing infrastructure. In this study we present a reimplementation as well as an extension of a technique using indicator vectors to compute and visualize similarities between sets of nucleotide sequences. We have a flexible and easy to use python program relying on standard and open-source libraries. Our tool allows analysis of very large complement of sequences using code parallelization, as well as by providing routines to split a computational task in smaller and manageable subtasks whose results are then merged. This implementation also facilitates adding new sequences into an indicator vector-based representation without re-computing the whole set. The efficient synthesis of data into knowledge is no trivial matter given the size and rapid growth of biological sequence databases. Based on previous results regarding the properties of indicator vectors, the open-source approach proposed here efficiently and flexibly supports comparative analysis of genetic diversity at a large scale. Our software is freely available at: https://github.com/WandrilleD/pyKleeBarcode.

## Introduction

The study of biological evolution through comparing linear sequences from different species, now known as molecular phylogeny, was first proposed by Crick in a 1957 lecture [1, 2]. The passage that heralds the coming of molecular phylogeny is clear and to the point:

“Biologists should realize that before long we shall have a subject which might be called ’protein taxonomy’ -the study of the amino acid sequences of the proteins of an organism and the comparison of them between species. It can be argued that these sequences are the most delicate expression possible of the phenotype of an organism and that vast amounts of evolutionary information may be hidden away within them.”

Linear sequence analysis of proteins from different species combined with paleontology to estimate chronological time of their divergence gave rise to the concept of a ‘molecular clock’ [3, 4], which proposes a constant number of amino acid changes per year in chronological time [5]. Directly inspired by the molecular clock, the neutral theory of evolution proposes that many sequence changes in evolution result from population effects such as drift, minor intermittent selection, and bottlenecks [6, 7]. The subsequent advent and development of DNA sequencing led Kimura to explicitly extend the neutral theory to DNA sequences with the reasonable hypothesis that synonymous mutations were neutral [8]. While the neutrality of synonymous sequences is not 100% valid, it is a useful first order approximation in many systems [9].

Woese and colleagues pioneered the use of small subunit ribosomal RNAs to construct phylogenies that encompass all of cellular life [10, 11]. SSU RNAs have two important features in this regard: a) Presence and sufficient similarity in all cellular life to allow them to be aligned and compared. b) Abundance and relative stability, facilitating their purification and characterization. Molecular methods including in situ hybridization and PCR allowed the characterization of SSU RNAs and the genes encoding them in situ including uncultured and unculturable organisms [12].

Mitochondrial genomes are small in size, lack recombination, and are technically straightforward in terms of molecular biology because their copy number is approximately a thousand times greater than nuclear genes. Mitochondrial analysis both confirmed and refined other methods for species and population identification, especially among animals [13, 14] but with certain caveats among other Eukaryotes, e.g., plants and fungi, as well [15]. Hebert and colleagues proposed that a partial segment of the mitochondrial genome agreed upon by the community could universalize and democratize species identification [16, 17]. The particular mitochondrial sequence that has become the most widely used, a 648 base pair (bp) segment of the gene encoding mitochondrial cytochrome c oxidase subunit I (COI), became most widely adopted because reliable primers and methods useful for both vertebrates and invertebrates were adopted by a critical mass of the community. Intrinsically, there is nothing special about the mtCOI DNA barcode region compared to other protein-encoding regions of the mitochondrial genome [18].

The Barcode Of Life Database (BOLD) is an open access compilation including mtCOI barcode data from approximately five million individuals that collectively cover a great deal of the extant animal kingdom [19]. The BOLD approach is a different approach in the world of sequence analysis because it is a small proportion of the genome (e.g., in humans less than one millionth of the total genome) but is available from many different individuals in many species. For many animal species DNA barcodes are the only sequence information available. The use of BOLD was first limited to species identification. The broad but not deep nature of barcode data can be used for more than identification, they can support phylogenic reconstructions and evolutionary models [20].

A method of choice to extract and recapitulate the evolutionary information contained in a set of homologous sequences is to construct a phylogenetic tree based on the of likelihood of a substitution model. These methods have benefitted from numerous advances in the past decades, but the explosion of the number of possible tree topologies as the number of leaves increase—(2n-5)!!, unrooted topologies for n leaves–still makes the reconstruction of phylogenetic trees with tens of thousands of sequences using likelihood methods daunting (although there exist strategies to improve the scalability using phylogenetic placement, see [21]). Aside from computational consideration, one has to consider cases where the evolutionary history of sequences does not follow a strictly tree-like pattern because of horizontal and modular evolution [22].

Sirovich and colleagues developed an innovative approach to allow multiple DNA sequences both within and among many animal species to be compared such that the overall structure of animal life within and among species can be visualized [23, 24]. Their method relies on a contrastive approach to build indicator vectors which recapitulates the genetic information of a sequence, or a group of sequence, which can then be used to probe the genetic diversity, or closedness of the taxonomic groups they represent, for example in the form of a similarity matrix called the structure matrix. Visualization of the structure matrix has been a valuable part of a number of publications [18, 25–31]. However, the indicator vector and structure matrix approach for the comparative study of DNA sequences has not yet been utilized to its full potential. The present work was originally motivated to make the prior work of Sirovich and colleagues more accessible as the implementation of software to compute the indicator vectors and Klee diagrams has been limited to the original code released with [23] (currently available at https://phe.rockefeller.edu/barcode/klee.php), which runs on an older version of a proprietary commercial software, Matlab2009a, a not only unmaintained version, but whose documentation is no longer available on the developer’s website. We used the opportunity of a re-implementation to introduce significant extensions to the original methods, in particular to accommodate a variable number of sequences per species, and provide a more flexible interface to the computational steps involved in going from a multiple sequence alignment to a structure matrix. Refactoring the process into separate steps allowed an efficient and scalable parallelization of the pipeline, as well as adding flexibility. In the previous Matlab implementation updating a structure matrix with one or several new sequences required recomputing it entirely. Separation of different steps in the implementation grants additional flexibility, both computationally and conceptually. Real-life datasets from BOLD (The Barcode Of Life Database) are used to illustrate the new implementation and in places contrast it with the published Matlab-based method.

## Methods

### Derivation of the original method’s formula

After a series of pre-processing steps, which are detailed in the result section of this manuscript as well as in [24], the multiple sequence alignment is split in several sequence sets.

A set of sequence *i* is represented as matrix *s*_*i*_ of size *m*×*s*⋅4, where *m* is the number of sequences in the sequence set and *s* is the sequence size, in nucleotides.

Then, the indicator vector of sequence set *i* is computed as the eigenvector with the largest positive eigenvalue of matrix:

$$s_{i}{s_{i}}^{T}-\frac{1}{N-1}\sum_{i\neq j}s_{j}{s_{j}}^{T}$$,

where *N* is the number of sequence sets, as defined in equation 15 of [24]. Note that this formulation presumes that all sequence set contains the same number of sequences. We begin by writing:

$$S_{i}=s_{i}{s_{i}}^{T}$$

such that the previous equation can be written $S_{i}-\frac{1}{N-1}\sum_{i\neq j}S_{j}$

This formula can be rewritten as:

$$\frac{N}{N-1}S_{i}-\frac{1}{N-1}\sum_{j}S_{j}$$

Which makes it evident that once the $\sum_{j}S_{j}$ matrix has been computed; the indicator vector of a sequence set can be computed independently from the other sequences. Additionally, the original method only considers the case were the number of sequences per set, noted *m*, is the same for each set. We generalize this approach to any specific number of sequences per set, noted m_i_ for sequence set *i* by normalizing the S_i_ matrices by m_i_, such that the previous equation becomes:

$$\frac{N}{N-1}\frac{S_{i}}{m_{i}}-\frac{1}{N-1}\sum_{j}\frac{S_{j}}{m_{j}}$$

Given its role of contrasting a given sequence set against all others, we call the matrix $\sum_{j}\frac{S_{j}}{m_{j}}$ the reference matrix.

Finally, it can be remarked that the computation of all, or parts of, the structure matrix depends only on the indicator vectors of the sequences of interest.

Consequently, we describe the method of [24] as three consecutive steps:

1. Computation of the reference matrix from all sequence sets
2. Computation of an indicator vector for each sequence set independently
3. Computation of the structure matrix from the indicator vectors of all sequence sets

We detail each of these steps in the results section.

### Mammalian sequences dataset

In order to test and demonstrate our method, we accessed the Barcode Of Life Database [19] for primary COI5-P sequences of mammals. Out of the >100,000 mammalian sequences, we selected a limited set, in order to facilitate results analysis and interpretation.

This set was built such that each of the following mammalian taxon is represented: Afrotheria, Artiodactyla, Carnivora, Chiroptera, Dermoptera, Eulipotyphla, Glires, Metatheria, Perissodactyla, Pholidota, Primates, Prototheria, Scandentia, Xenarthra.

To ensure this, we randomly selected a maximum of 40 species per taxon, and 3 sequences per species, except for Prototheria, Pholidota, Scandentia and Dermoptera where the low number of sampled species and sequences caused us to take all available sequences (resp. 3, 7, 9, and 1 species, and 7, 21, 16, and 3 sequences). The resulting set is composed of 1049 sequences spread among 354 species.

The retrieved sequences were then aligned using MAFFT [32, 33]. The sequence alignment extremities were then trimmed to avoid the spurious gaps resulting from differences in the size of the sequenced fragment. The resulting multiple sequence alignment presented no gaps (-), no missing base (N) characters, and 0.007% ambiguous IUPAC characters (Y,R,W,etc.).

### Impact of the reference matrix on the indicator vector and structure matrix

One of the new possibilities offered by our re-implementation is the possibility to compute the indicator vectors of sequences which are absent from the reference matrix. This can be useful, for instance, if a new sequence were just acquired, because it could be integrated to the structure matrix without having to re-compute it entirely.

In order to evaluate the impact of an incomplete reference matrix, we devised a number of experiments where we compute the indicator vectors and structure matrix of our mammalian dataset sequences with a reference matrix computed of a limited set of sequences. We then compared these with the indicator vectors and structure matrix obtained with a complete reference matrix.

The initial experiments were “primate-only”: reference matrix with primate sequences only (120 primate sequences); “no-Laurasiatheria”: reference matrix with Laurasiatherian sequences missing (488 non-Laurasiatherian sequences); and “missingXX%”: random percentages of sequences missing.

For the “missingXX%”, we tested percentages from 10 to 90 percent (by increments of 10%). We performed 10 replicates per condition to assess the variability in the results.

## Results

**Fig 1:**
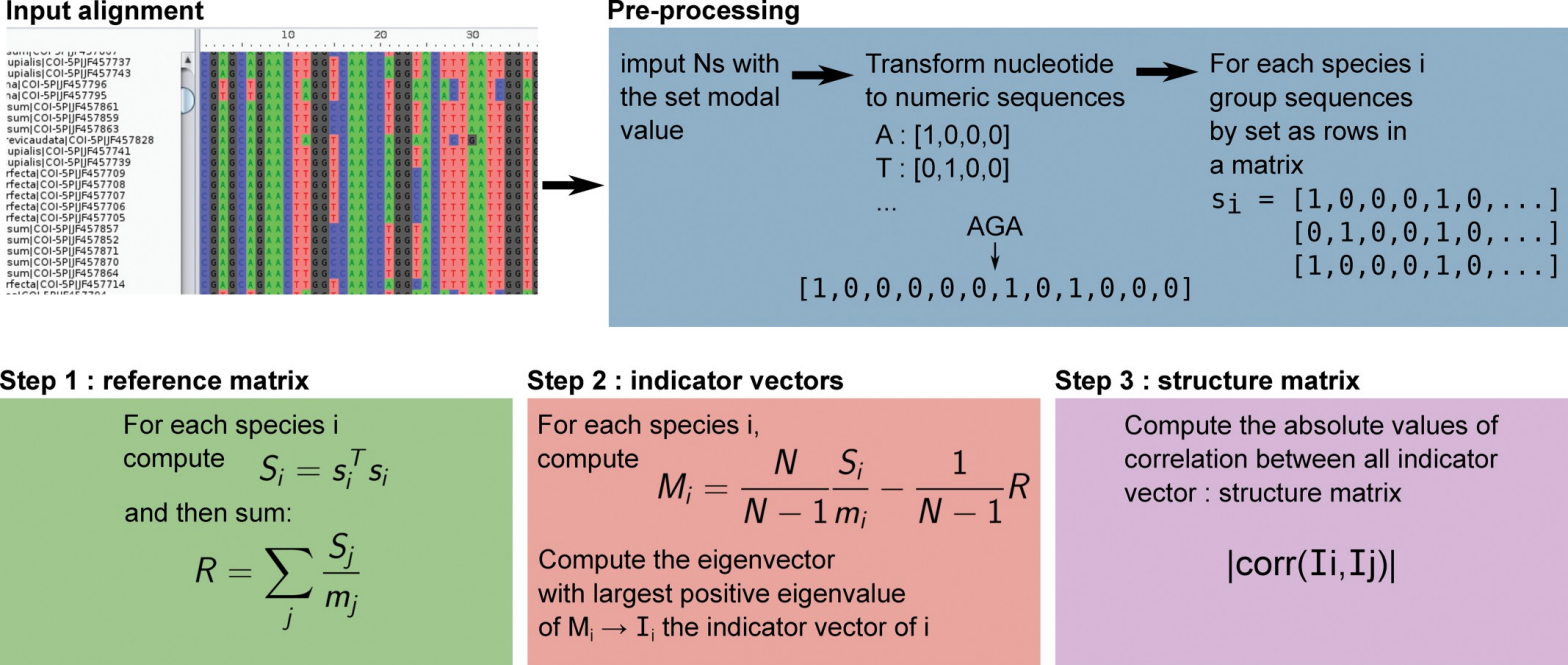
Schematic description of pyKleeBarcode pre-processing and steps.

We have implemented each of the following steps as an independent piece of code with its specific parallelization scheme, as well as in a single executable regrouping all three steps for convenience. All implementations were done in python3 using core modules and the publicly accessible libraries numpy, scipy, matplotlib, mpi4py. The source code is available at https://github.com/WandrilleD/pyKleeBarcode, complete with a documentation, tests, and example of usage with toy datasets. Fig 1 gives a schematic overview of the steps involved in the process of going from an input multiple sequence alignment to a structure matrix.

PyKleeBarcode takes as input a multiple sequence alignment in the fasta format, whose sequences are grouped into sequences sets, most frequently corresponding to species.

The grouping of individual sequences into sets is deduced from part of the sequence name in the input fasta file, or via an association table provided using an optional argument to the script. Note that a sequence set may contain a single sequence. Also, note that while the original method from [24] enforces the constraint that each sequence set has the same number of sequences, pyKleeBarcode allows sequence sets of different sizes.

### Sequence alignment pre-processing steps

To compute the structure matrix from a multiple sequence alignment, the DNA sequences are first pre-processed and transformed to vectors of four numbers. The pre-processing consists in a tentative imputation of the ambiguous N characters: any N character is replaced by the modal nucleotide at this position among its sequence set (typically, sequences of the same species in the multiple sequence alignment), unless that modal value is the gap character (“-”), or there are multiple non-gap character modal values, in which case the value stays N at this position. This pre-processing step is unchanged from the original method of [24]. Then the sequences are transformed to numerical vectors where each nucleotide is represented using four numbers, such that A corresponds to [1,0,0,0], C to [0,1,0,0], G to [0,0,1,0], and T to [0,0,0,1]), and the gap character “-" to [0,0,0,0] (formula 1 and 2 of [22]), additionally we have added support for ambiguous IUPAC characters, which corresponds to a vector with the different possibilities having the same weights and summing to 1 (*e*.*g*., B may correspond to C,G, or T and is represented as [0,1/3,1/3,1/3]).

The numerical representations of each sequence are then grouped by set into matrices where each row is the numerical vector of a single sequence. Thus, sequence set *i* is represented as matrix s_i_ of size *m*×*s*⋅4, where *m* is the number of sequences in the sequence set and *s* is the sequence size, in nucleotides.

This series of pre-processing steps are represented in Fig 1. They are performed either once, or at the beginning of step 1 and step 2 when these are executed as independent executables.

### Step1: Reference matrix computation

This step corresponds to the computation of a matrix representation of the diversity of a DNA sequence across several individuals or sets of individuals (typically, species) that will serve as a reference against which individual sequence will later be contrasted (see step 2).After sequences have been transformed into numerical vectors and grouped into matrices s_i_ as described in the pre-processing steps above and the top part of Fig 1, the reference matrix R is obtained by computing, for each sequence set i:

$$S_{i}=s_{i}{s_{i}}^{T}$$

And then summing all S_i_ matrices, normalized by the number of sequences in each set m_i_:

$$R=\sum_{j}\frac{S_{j}}{m_{j}}$$

See the Method section detail how we derive this formulation from the original formulas of [24].

Reference matrix computation has a time complexity of *O*(*Ns*^2^) and a memory complexity of $O(\sqrt{N}s^{2})$.

When executed as an independent script, it takes as input a multiple sequence alignment in the fasta format, and as output the reference matrix in a simple binary format, chosen for its read/write performance.

The script is parallelized using MPI and thus it can be deployed on multiple CPU architectures. Briefly, the different sequences sets are split between the different processes, each computing a local reference matrix, which are then combined before being written to a file.

Furthermore, we provide a utility script to merge pre-existing reference matrix together (provided they were computed on an independent set of sequences and that no grouping/species are shared). This allows one to update a previously obtained matrix with new sequences.

Additionally, it also permits the computation of a reference matrix to be subdivided in any number of subtasks, by simply splitting the input alignment, and subsequently merging the resulting matrices. This makes the computation of very large datasets tractable and easy to deploy on one or multiple HPC architecture.

### Step2: Indicator vector computation

This step corresponds to the computation of indicator vectors for each sequence or sets of sequences provided.

As per [24], the indicator vector of a sequence set is defined as the eigenvector with the largest positive eigenvalue of a matrix contrasting the sequence set against all other sequence sets. Using the definitions provided above, for sequence set i this matrix is computed as:

$$M_{i}=\frac{N}{N-1}\frac{S_{i}}{m_{i}}-\frac{1}{N-1}R$$

See the Method section detail how we derive this formulation from the original formulas of [24].

Indicator vectors computation has a time and a memory complexity of *O*(*Ns*^2^). When executed as an independent script, it takes as input a multiple sequence alignment in the fasta format, the reference matrix (as obtained from the previous step), and it outputs indicator vectors in a csv format file.

The computation of each individual indicator vector being independent from the rest, the parallelization of this script using MPI is fairly straightforward.

As with step1, it is possible to further split the computations in many subtasks by splitting the input alignment before invoking pyKleeBarcode. The subsequent merging of the resulting file is done by simple concatenation.

### Step3: Structure matrix computation

In this step a structure matrix, containing the correlation between the indicator vectors of different sets of sequences, is computed.

In pyKleeBarcode it may be computed either in one go as the inner product of in two fashions. Either in one go, following the formulation of [24], with a single multiplication of a matrix where each row is an indicator vector by its transpose, or in multiple steps where each step computes a line from the structure matrix.

The first option is faster—albeit both options have the same time complexity–but more memory-intensive because the whole structure matrix needs to be held in memory at once, while the second one is slower but requires less memory.

The switch between these two options is, by default, set at 5,000 indicator vectors, corresponding to about 800Mb of memory, and can be modified to suit the available resources.

Structure matrix computation has a time complexity of *O*(*Ns*) and a memory complexity of *O*(*N*^2^*s*) when the number of indicator vectors is small, and *O*(*Ns*) otherwise.

This step takes as input the set of indicator vectors, in csv format, for which to compute pair-wise correlations; and it outputs the structure matrix (in a binary format of its lower triangular portion).

While the previous steps could be parallelized and split in subtasks in a straightforward manner, the structure matrix computation matrix computation is a more complicated affair, because it must look at all pairs of indicator vectors.

Nevertheless, we have devised an algorithm, and provide the corresponding script, to update an existing structure matrix with new indicator vectors (and thus, new sequences).

In fact, as structure matrices grow quadratically in size with the number of sequence sets they represent (count about 38Gb for 100,000 sequence sets), the structure matrix file format has been explicitly devised with the goal of allowing an update without having to read, or even re-write the whole file; the new information is merely appended to it.

Overall, throughout our various benchmarks and experiments, Step2 has consistently been the step that took most of the computational time (usually between 80% and 90%). When using a single MPI process, pykleebarcode has a performance of the same order of magnitude as the original Matlab code (see S2 Fig); the usage of several MPI processes diminishes runtime due to the increased amount of computational resources (see S2 Fig).

### Impact of the reference matrix on the indicator vector and structure matrix

**Fig 2:**
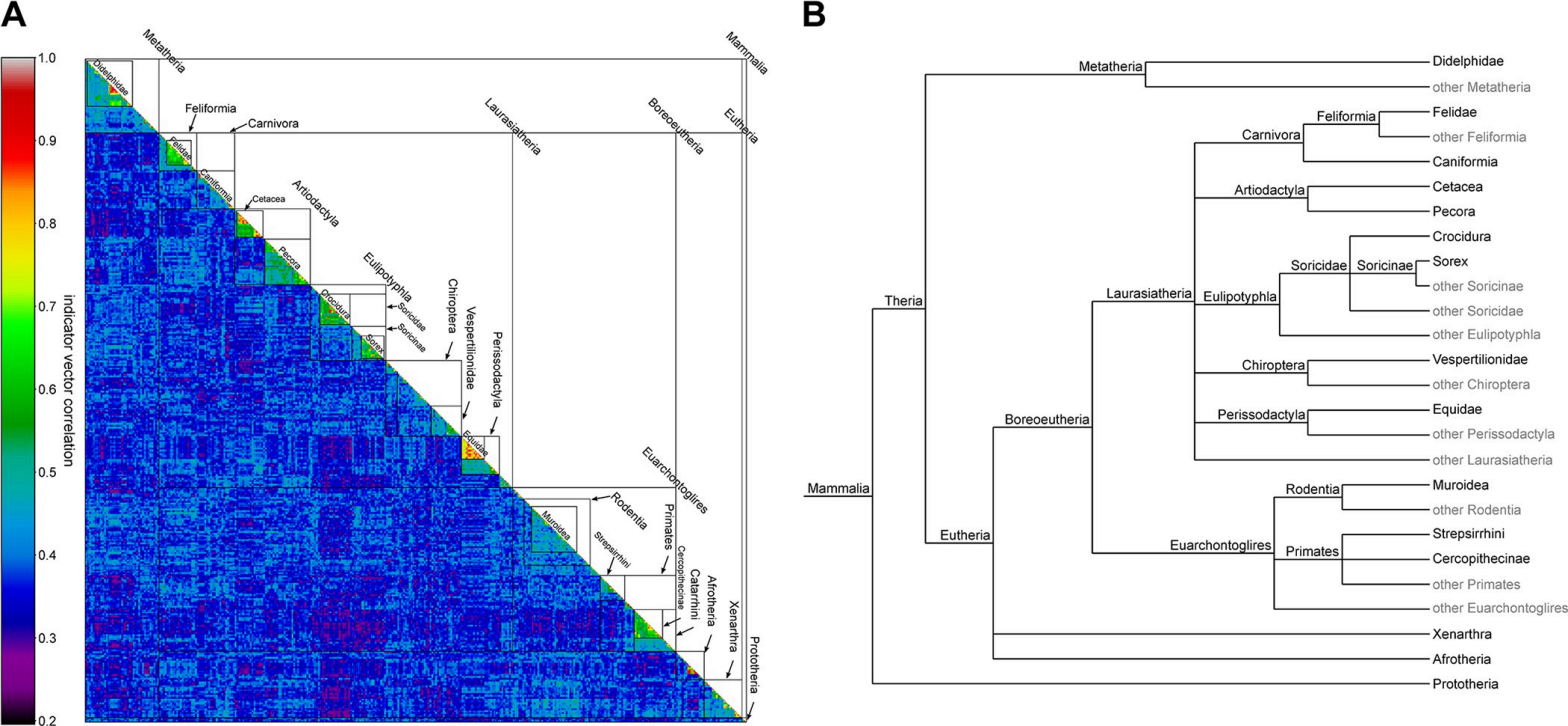
A. View of the structure matrix of the mammalian dataset and taxonomic structure of Mammalia. B. Phylogenetic tree structure of the taxonomic groups retrieved from NCBI taxonomy.

Fig 2A presents a view of the structure matrix of the mammalian dataset obtained with the whole data set as reference matrix. To help interpretation, rows and columns have been ordered and annotated with taxonomic groups retrieved from the NCBI taxonomy [34], whose hierarchical structure is displayed on Fig 2B. It should be noted that the ordering of groups inside the same taxonomic unit is arbitrary (e.g.: the appearance of Carnivora next to Artiodactyla in Laurasiatheria). In this structure matrix, it appears that the correlation of indicator vectors is higher among the sequences closely lower taxonomic unit (such as Equidae, or Pecora). However, between less closely related groups (ie, off the structure matrix diagonal) the correlation fluctuates between 0.2 and 0.4. This effect is related to the genetic saturation of the studied COI5-P sequence (see S3 Fig). For reference, steps 1, 2 and 3 took respectively 5, 30 and 1 seconds as well as 95, 162, and 92 megabytes, on an Intel i7-8665U CPU on this dataset of 1049 sequences of 384bp took (for the purpose of this test each sequence were kept separate as their own set).

To assess the effect of the impact of an incomplete reference matrix on the indicator vectors and structure matrix we designed three experiments as described in the methods section. The “primates-only” experiment serves to assess a case of extrapolation: computing indicator vectors on sequences which are outgroups to the ones included in the reference matrix. In contrast, the “no-Laurasiatheria” explores a case of interpolation, albeit one an extreme one where an entire super-order is missing. Finally, the “missingXX%” experiment addresses mixed cases of extrapolation and interpolation but where no large taxonomic group is missing.

**Fig 3:**
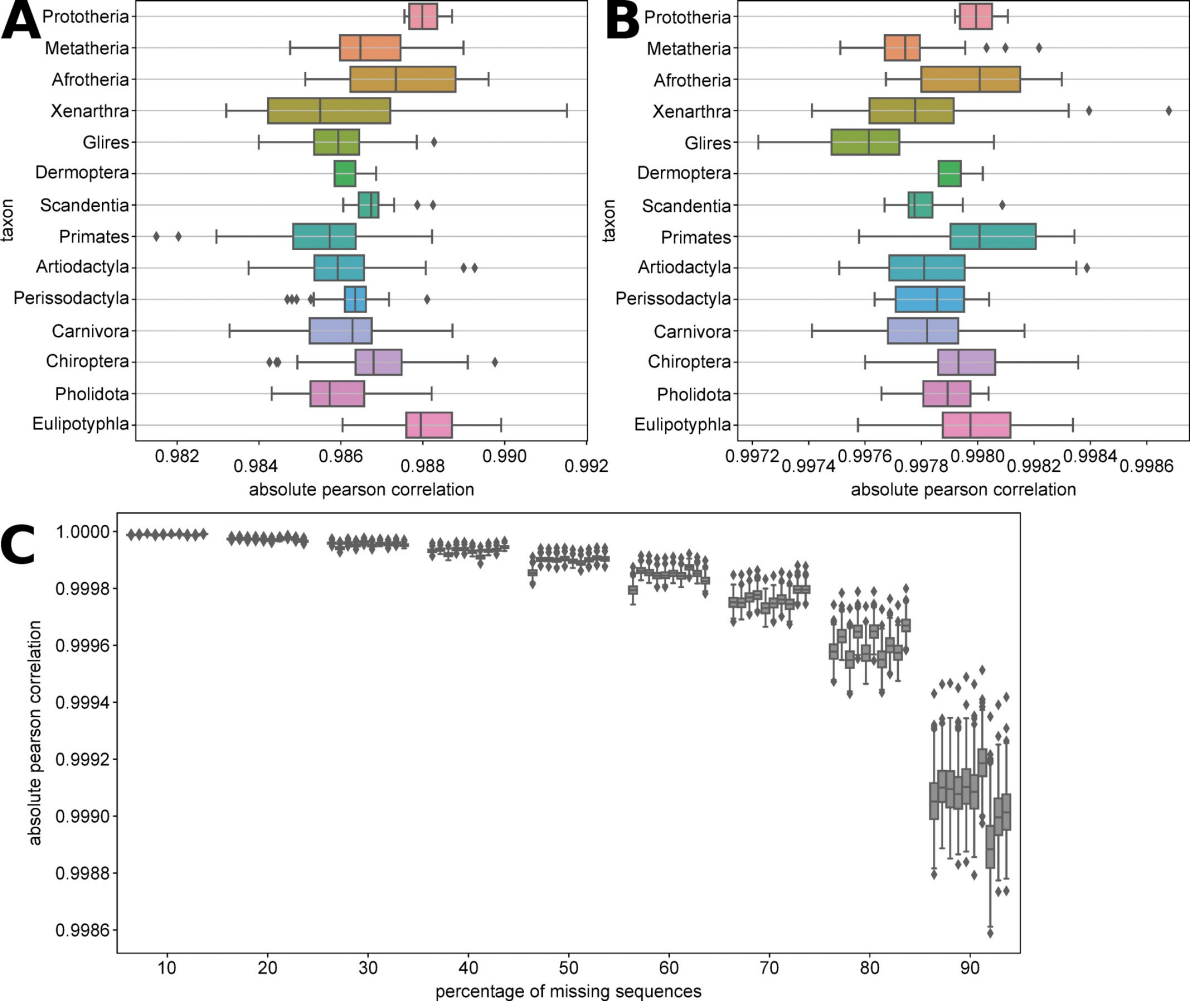
A. Absolute Pearson correlation between indicator vectors obtained on the complete and the primate-only reference matrices. B. Absolute Pearson correlation between indicator vectors obtained on the complete and the no-Laurasiatheria reference matrices. C. Evolution of absolute Pearson correlation between indicator vectors obtained on the complete and a limited reference matrix with an increasing percentage of randomly missing sequences. Each boxplot corresponds to a single random replicate.

**Fig 4:**
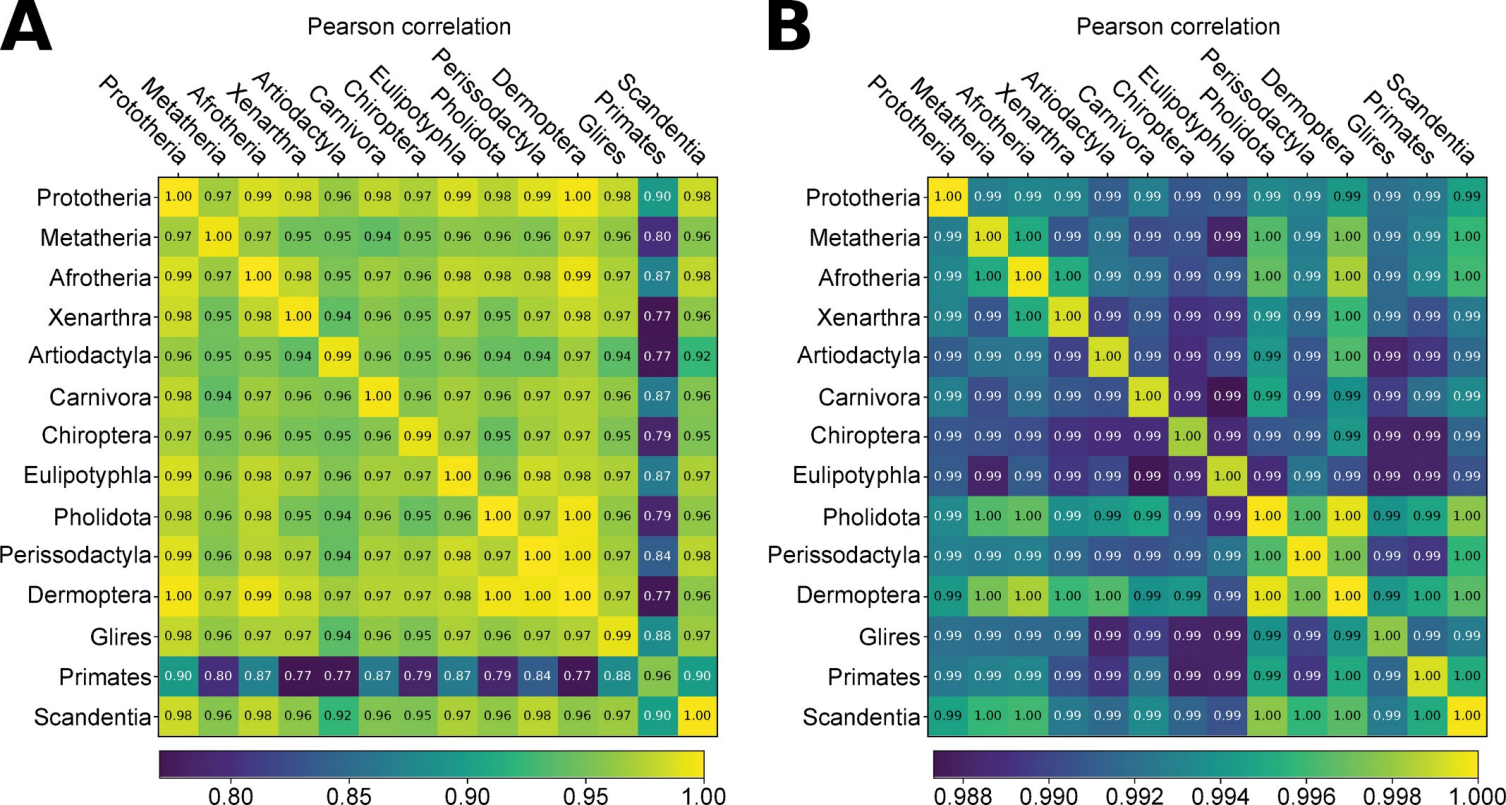
Pearson correlation of the structure matrices with the reference one, grouped by main taxa. A. comparison of the primates-only structure matrix and the reference one. B. comparison of the no-Laurasiatheria structure matrix and the reference one.

Fig 3A presents the absolute Pearson correlation values between the indicator vectors obtained with a reference matrix containing all sequences versus a reference matrix containing the primate sequences only (“primates-only” experiment). All values are above 0.980, despite the “primates-only” reference matrix containing less than 12% (120/1049) of sequences, and all coming from the same subgroup of the data: primates in this instance. Interestingly, it appears the indicator vector of primate sequences themselves are among the most impacted as it presents the lowest average correlation value of all the main taxa as well as contains the vector with the overall lowest correlation value. Fig 4A reinforces this impression, as it shows that values in the structure matrix obtained from primates-only reference are highly correlated with the ones obtained with the full reference, but with a notable decrease when it comes to values describing the similarity between primates and other groups. This is suggestive of a behaviour where the effect of extrapolation (here, computing a structure matrix with some non-primates on a reference matrix containing primates sequences only) mostly affects the displayed relationship between the extrapolated sequences and the sequences used for building the reference matrix, rather than between the different extrapolated sequences.

The results of the “no-Laurasiatheria” experiment, presented in Fig 3B, also show very good correlations with the full reference matrix, with all values being above 0.997. Regarding a pattern where the sequences included in the reference matrix (i.e., the non-laurasiatherians) are on average lower than the ones excluded (i.e., laurasiatherians), while a Mann-Whitney U yields a small p-value (<10^−12^), the actual difference in median is only about 0.0001. Fig 4B also does not exhibit a pattern which specifically differentiate Laurasiatherians from others when comparing the structure matrices. Thus, it would seem that "interpolation” of new sequences in a structure matrix is not specifically affect the new sequences, even when they are from a group which is entirely missing from the reference matrix.

Fig 3C shows the results of the “missingXX%” experiment, where sequences are missing at random from the reference matrix. We see an expected pattern of general decrease in absolute correlation as the percentage of missing sequences increases, albeit all values stay above 0.9998 until 60% of sequences are missing and remain above 0.998 even when 90% of the sequences are missing. We also observe that the variation between replicates is limited, showing that this trend is robust to random sampling effects.

## Discussion

PyKleeBarcode builds on the previous works on indicator vectors, and allows several new possibilities, such as an extended handling of ambiguous nucleotide character, but more importantly the ability to have differences in the number of sequences included per group, as well as the possibility of efficient integration of new results onto a pre-existing structure matrix—something hitherto not possible. As our experiments have shown, this has only minimal impact on the computed indicator vectors and similarities between sequences, except in the direst of scenarios, such as in the primates-only experiment, or when the percentage of new sequences is above 50%. Consequently, our method is appropriate to integrate a growing set of sequences, continuously integrating new specimens to see where they fit among the existing structure, which could only need to be entirely recomputed for major version releases for example.

Considering PyKleeBarcode, and the indicator vectors approach in general, in the context of other methods used to extract insight from homologous sequences, it first presumes that a multiple sequence alignment has already been obtained, using MAFFT [32, 33] for example, in order to establish homology nucleotide by nucleotide. Thus, in an analysis pipeline it would come after tools used to establish a distance-based homology between a single sequence and among a larger database, such as BLAST [35], mapping tools such as bwa [36] or minimap2 [37], or dedicated software and databases (such as SiLiX [38] and HOGENOM [39] for instance), which are not the most appropriate or efficient to compute all pairwise distances between already aligned sequences.

As we noted above our approach can be contrasted with phylogenetic tree reconstruction methods: pyKleeBarcode does not create an explicit evolutionary scenario, but only a distance matrix. Consequently, pyKleeBarcode is robust to cases where sequences do not follow a strictly tree-like evolutionary scenario and is not hindered by the unfavorable combinatorics of the number of possible tree topologies. In contrast, likelihood-based tree reconstruction methods must constantly engage in compromises between result approximation and runtime to completion.

Furthermore, the distance matrix produced by pyKleeBarcode may be used as the basis to construct a tree, using a distance-based method such as Neighbor-Joining [40] or UPGMA [41]. PyKleeBarcode may be used in complement with the results of likelihood-based methods to point out discrepancies that suggest reticulated evolution or convergences.

The indicator vector approach is most closely related with pairwise-distance methods and shares some of their limitations such as a sensitivity to genetic saturation. The main specificity of our approach relies on the elegant approach of [23] to conserve the richness of sequence diversity information when grouping sequences at various taxonomic levels rather than relying on a single consensus sequence per group.

Some properties of indicator vectors are still unclear. For example, the contrastive nature of the approach should make them robust against heterogeneities in the mutation patterns of sequences, but this remains to be tested. Similarly, it would be of interest to investigate potential biases introduced by sampling differences between studied clades.

With pyKleeBarcode we propose a flexible and up-to-date interface to compute indicator vectors and structure matrix from a multiple sequence alignment in a manner, adapted to the size of modern datasets and large computer infrastructures.

We anticipate that pyKleeBarcode will help undertake deeper analyses of biological sequence databases, including BOLD, and allow new insights into large scale features of extant life.

## Supporting information

S1 File Supporting information for “PyKleeBarcode: Enabling representation of the whole animal kingdom in information space “Appendix A: Benchmarking of pykleeBarcode against the previous Matlab implementation, and Appendix B: Investigating the saturation of the COI5-P among mammals evolution.
(PDF)

S1 Fig Evolution of execution time (A) and peak RAM usage (B) for the computation of a structure matrix with the number of DNA sequences for different implementations.
(TIF)

S2 Fig Estimation of the speedup of pykleeBarcode achieved with 4 MPI processes. The red line represents the average on a rolling window of 20 points.
(TIF)

S3 Fig Evolution of COI5-P hamming distances with divergence time among mammals. The red line corresponds to a rolling average of a 20 MYA window.
(TIF)
