# Supplementary material for: PyKleeBarcode: Enabling representation of the whole animal kingdom in information space
Source: PLoS One. 2023 Jun 2;18(6):e0286314. doi: 10.1371/journal.pone.0286314 (PMC10237437; doi:10.1371/journal.pone.0286314)
Supplement: S1 File — Supporting information for “PyKleeBarcode: Enabling representation of the whole animal kingdom in information space “Appendix A: Benchmarking of pykleeBarcode against the previous Matlab implementation, and Appendix B: Investigating the saturation of the COI5-P among mammals evolution. (PDF) [file pone.0286314.s001.pdf]

Supporting Information for

"PyKleeBarcode: Enabling representation of the whole animal kingdom in information space "

Wandrille Duchemin, David S. Thaler

Appendix A : Benchmarking of pykleeBarcode against the previous Matlab implementation

This analysis reports on the performance of two implementations using a single multithreaded CPU with an increasing number of sequences to process.

Material & Method

Using the 1049 mammal COI5-P sequences alignment presented above, we generated multiple sequence alignments containing from 100 to 5000 sequences by increments of 25 sequences. For alignments with more than 1049 sequences, we extended the initial alignment by randomly duplicating some sequences and adding a random mutation to their copy. For each alignment, we ran the python and Matlab scripts to go from input alignment to a structure matrix where each sequence is in its own sequence set. Computations were performed on 12 CPU threads of an AMD EPYC 7742 64-Core Processor, while monitoring execution time and peak RAM usage. Additionally, the python scripts were also run with 4 MPI tasks (with 12 thread each on the same CPU).

Of importance is to note that the Matlab script was not run on the unmaintained Matlab2009a for which we could not achieve a working computational environment. Instead, we used Matlab2021b for which the code seemed to run its course without error, albeit we cannot guarantee the exactitude of its results.

Results

When it comes to execution time with a single task, the Matlab and python implementation show comparable performances. Although python implementation is showing a clear advantage for low sequence counts, this is likely due to differential startup time between the two languages and indeed this difference decreases as the computational load increases, with the number of sequences. Peak RAM usage follows a similar pattern however here the initial difference of about 1Gb does not disappear. The speedup resulting from running the python implementation with 4 MPI tasks goes from a factor 2 for a number of sequences under 500, to plateau around a 3.3 factor when the number of sequences is above 1000. This also results in a moderate, but non negligible, memory overhead (note that the memory reported corresponds to the sum of all MPI tasks usage). Unfortunately, the tool used to track memory usage is imprecise for MPI tasks; we recommend allowing each MPI task as much RAM as needed to complete the computation without parallelization.

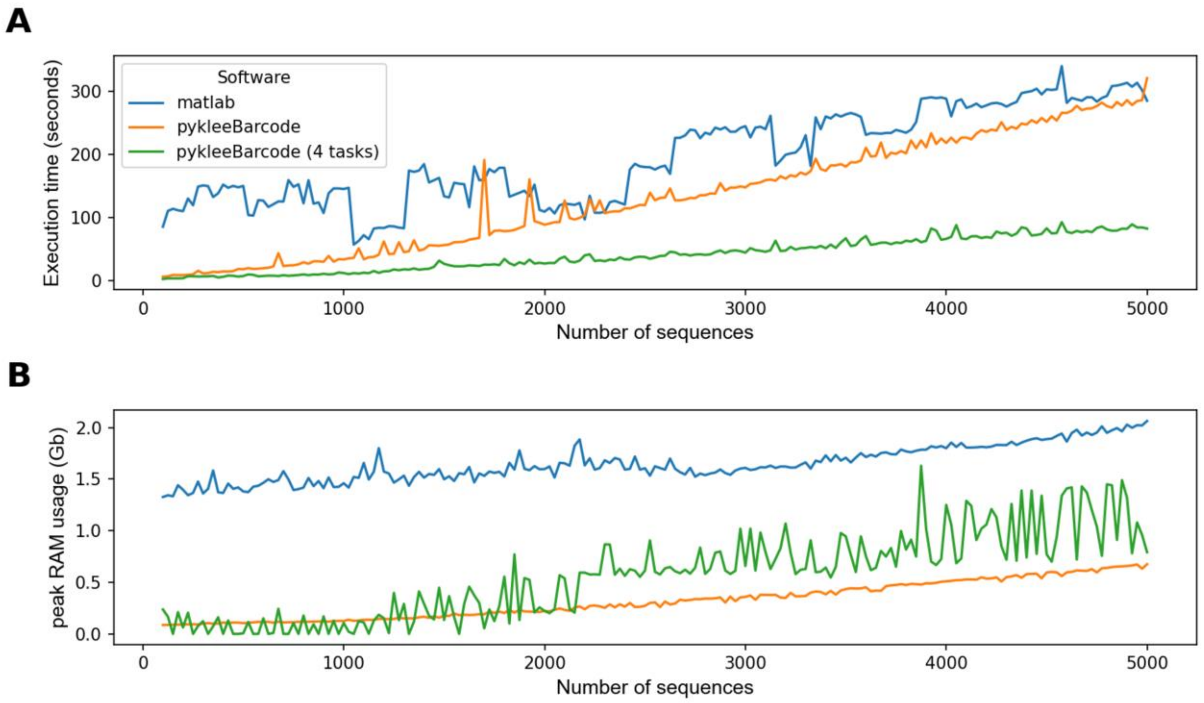

Fig. S1. Evolution of execution time (A) and peak RAM usage (B) for the computation of a structure matrix with the number of DNA sequences for different implementations.

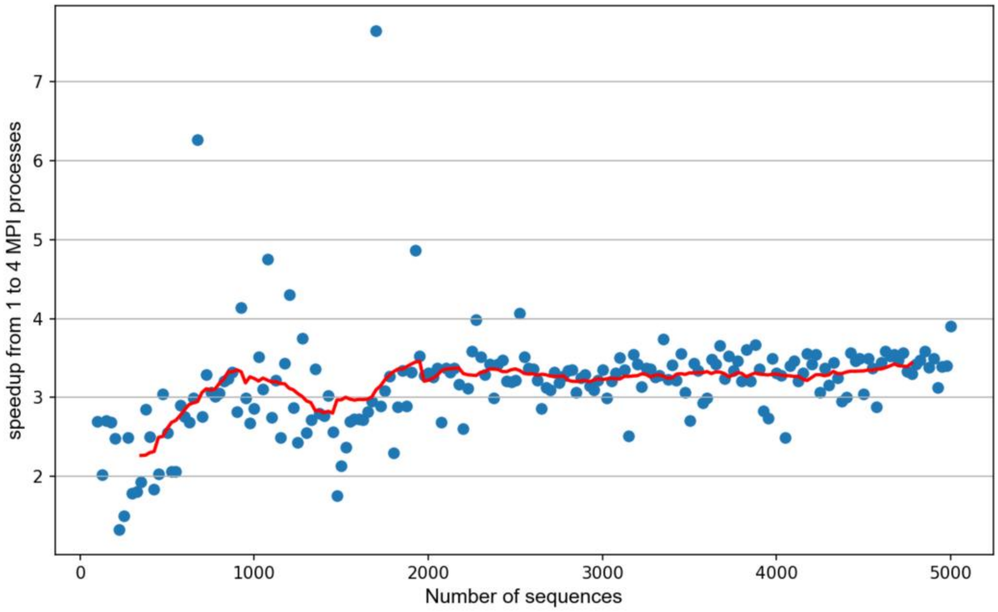

Fig. S2. Estimation of the speedup of pykleeBarcode achieved with 4 MPI processes. The red line represents the average on a rolling window of 20 points.

Appendix B : Investigating the saturation of the COI5-P among mammals evolution

Material & Method

To assess the patterns of mutation accumulation over time in mammal COI5-P sequences alignment presented above, we computed the pairwise hamming distance between the consensus sequences of each of the 354 species. Estimates of divergence between all pairs of species were retrieved from the timetree.org resource [1, accessed March 2023].

Results

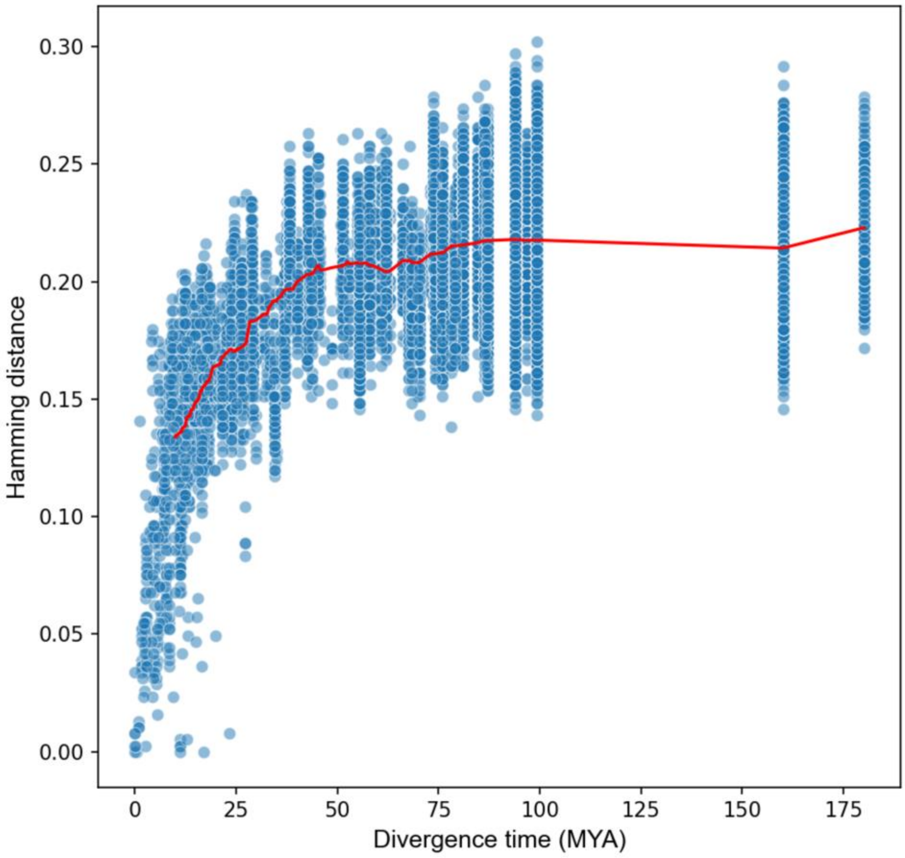

Fig S3. Evolution of COI5-P Hamming distances with divergence time among mammals. The red line corresponds to a rolling average of a 20 MYA window.

The relationship between the hamming distance between mammals COI5P sequences and estimated divergence time shown Fig S3 presents a clear pattern of genetic saturation, which after an initial proportional phase reaches a plateau.

Discussion

This observed saturation effect of the COI5P explains how the structure matrix based on this data is able to display the differences between closely related lineages but seems to show essentially random noise between distantly related lineages. Indeed, Fig S3 Reveal that after roughly 40 million years the accumulation of substitutions does not increase the distance between sequences.

References

1. Kumar S, Suleski M, Craig JM, Kasprowicz AE, Sanderford M, Li M, Stecher G, Hedges SB. TimeTree 5: An Expanded Resource for Species Divergence Times. Mol Biol Evol. 2022 Aug 6;39(8):msac174
