# Supplementary figures and images for: PyKleeBarcode: Enabling representation of the whole animal kingdom in information space
Source: PLoS One. 2023 Jun 2;18(6):e0286314. doi: 10.1371/journal.pone.0286314 (PMC10237437; doi:10.1371/journal.pone.0286314)

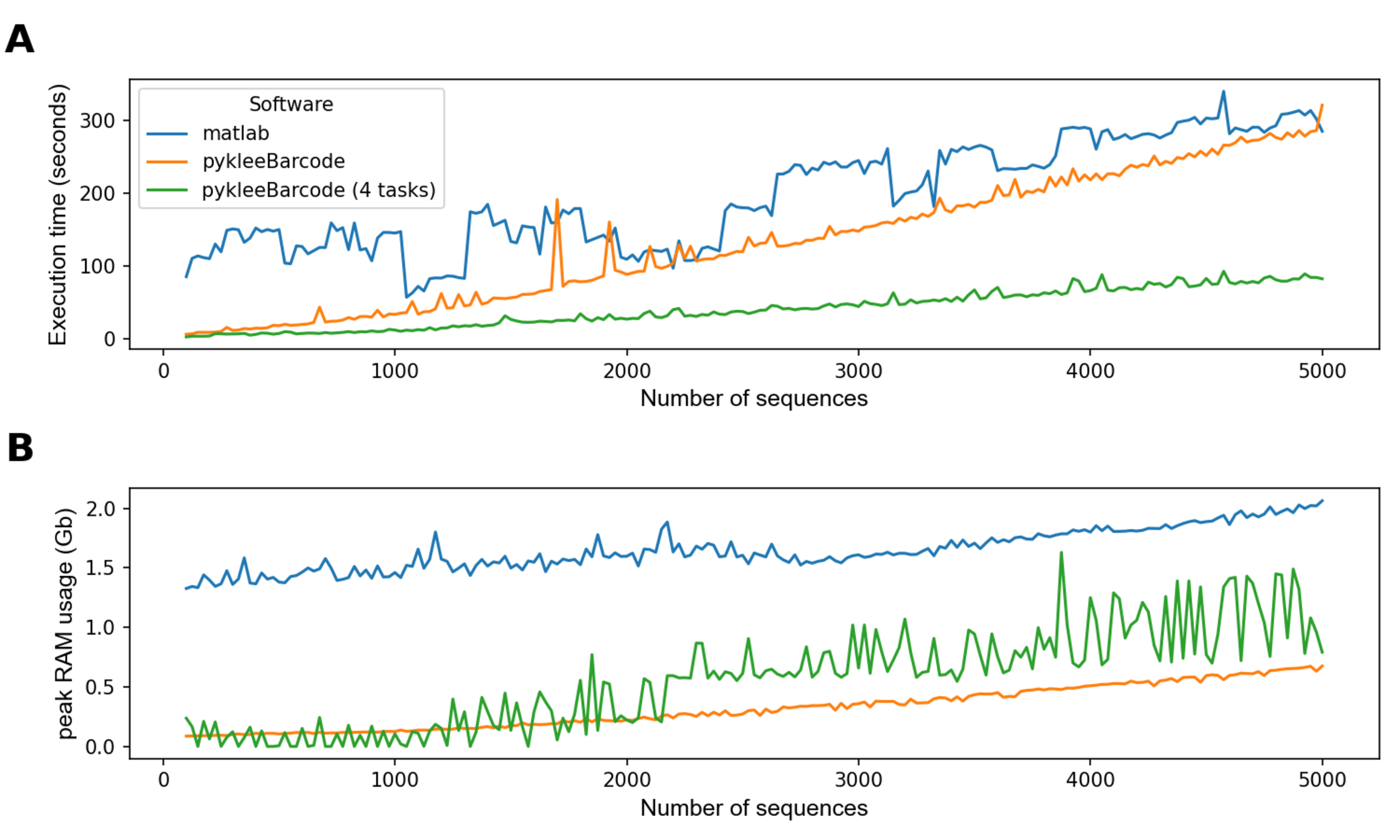

Supplement: S1 Fig — Evolution of execution time (A) and peak RAM usage (B) for the computation of a structure matrix with the number of DNA sequences for different implementations. (TIF) [file pone.0286314.s002.tif]

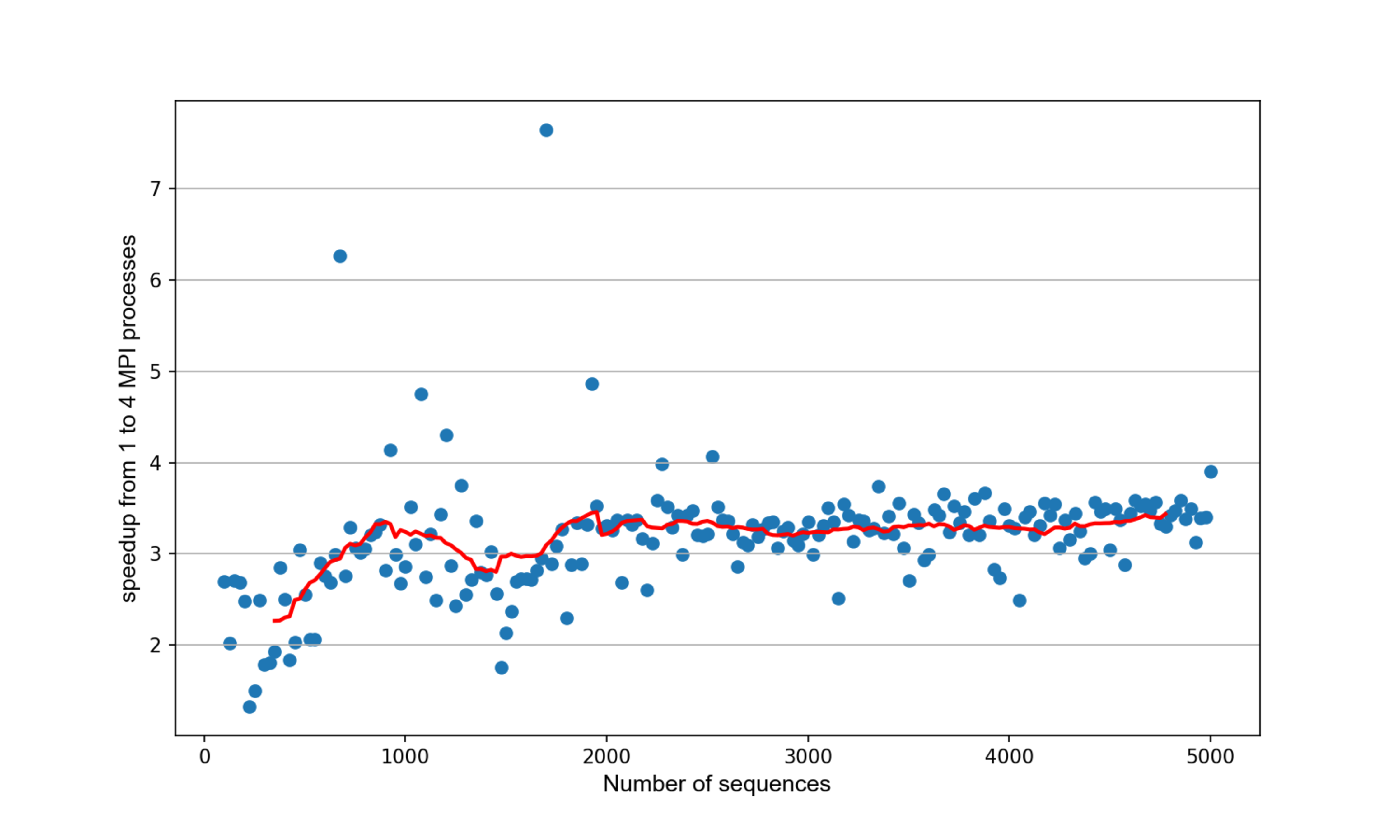

Supplement: S2 Fig — The red line represents the average on a rolling window of 20 points. (TIF) [file pone.0286314.s003.tif]

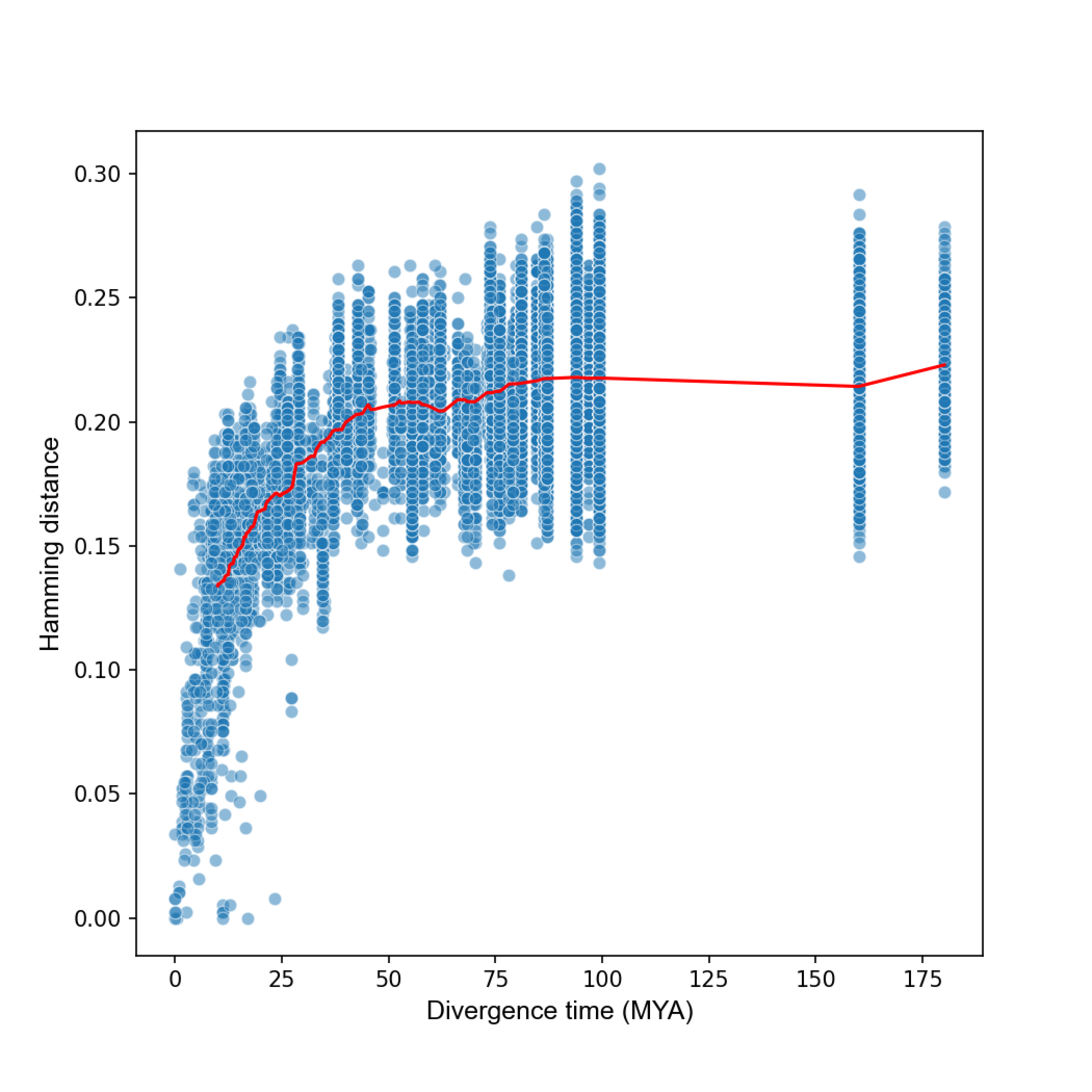

Supplement: S3 Fig — The red line corresponds to a rolling average of a 20 MYA window. (TIF) [file pone.0286314.s004.tif]
